# P341: Resistance factors to standards and procedures quality of care pplication in the republic of guinea

**DOI:** 10.1186/2047-2994-2-S1-P341

**Published:** 2013-06-20

**Authors:** MM Diallo, S Conde, BK Diallo, T Diallo, AV Dembele

**Affiliations:** 1 Focal Point RIPAQS Guinea, Obstetrician Gynecologist Ignace Deen NH, Conakry, Guinea; 2 DNEHS / MSHP, Conakry, Guinea; 3 Physician Standards and Procedures section DNEHS, Conakry, Guinea; 4 Ignace Deen NH, Conakry, Guinea

## Introduction

The study was organized around individual and collective factors of resistance associated with the implementation of the requirements and recommendations of standards and quality assurance standards of care in health facilities selected.

## Objectives

To determine the factors influencing the implementation and dissemination of quality standards and procedures of the professional practices of care in health facilities in Guinea.

## Methods

This cross-sectional study using the interview technique. It ran from a questionnaire on the operational side of the organization of the quality of care in health facilities. On the pan of the sample, this study has identified nine health facilities corresponding to the three levels of the health pyramid. The structures were stratified by type of institution (CHU, CMC, CS), a draw was able to identify the structures corresponding to the sample to be studied. This is a sample having a size of 88 subjects distributed among the strata according to their size represented by the staff working in administrative services and maternity health facilities drawn.

## Results

The main results are: i) A little knowledge of good practice recommendations by staff, ii) SONU represent the training approach in quality, iii) low RBP applicability of quality of care (40%), iv) The application of the organizational constraints RBP is on average 50%, v) weaknesses of policy favoring surveys (20%) and vi) Low financing application of standards and procedures for care (41%).

## Conclusion

It is recommended to train staff on best practices and clinical laboratory revise regulations for the implementation of quality standards and procedures, develop tools for monitoring and evaluating the implementation of quality standards and procedures and develop action research to resolve service issues.

## Disclosure of interest

None declared

